# The Anti-Inflammatory Actions of Soluble Klotho in Brain Aging and Its Main Associated Diseases

**DOI:** 10.3390/ijms26178551

**Published:** 2025-09-03

**Authors:** Héctor E. López-Valdés, Martín Hernández-Lucas, Gustavo D. J. Rodríguez-Fabián, Nadia F. Esteban-Román, Roger Gutiérrez-Juárez, Isabel Arrieta-Cruz, Hilda Martínez-Coria

**Affiliations:** 1 Departamento de Fisiología, Facultad de Medicina, Universidad Nacional Autónoma de México, México City 04360, Mexico; helopezv@comunidad.unam.mx (H.E.L.-V.); martinhl24@outlook.com (M.H.-L.); gustavofab@ciencias.unam.mx (G.D.J.R.-F.); nadia.esteban.roman@gmail.com (N.F.E.-R.); 2 Departamento de Ciencias Biomédicas, Escuela de Medicina, Facultad de Estudios Superiores Zaragoza, Universidad Nacional Autónoma de México, México City 09230, Mexico; roger.gutierrez@zaragoza.unam.mx; 3 Departamento de Investigación Básica, División de Investigación, Instituto Nacional de Geriatría, Secretaría de Salud, México City 10200, Mexico; arrieta777@mail.com

**Keywords:** α-Klotho, neuroinflammation, aging, Alzheimer’s disease, Parkinson’s disease, ischemic stroke

## Abstract

The anti-aging protein α-Klotho has several therapeutic effects on different pathophysiological conditions, mainly its anti-inflammatory and antioxidant effects. Experimental evidence and observational studies suggest that there are several strategies to increase α-Klotho in the brain and enhance its beneficial effects, thus contributing to improving its neuroprotective and neuroplasticity mechanisms in brain aging, Alzheimer’s, Parkinson’s, and ischemic stroke diseases. In this article, we summarize the relevant information on α-Klotho, brain aging, Alzheimer’s, Parkinson’s, and ischemic stroke diseases and analyze the role of α-Klotho in each of these alterations, as well as the effect of physical exercise, exogenous application of α-klotho, and various drugs approved for different human diseases on α-Klotho production.

## 1. Introduction

Alzheimer’s disease (AD), Parkinson’s disease (PD), and focal ischemic stroke (FIS) are diseases whose incidence increases significantly during aging and are among those that have the greatest impact on brain health in older adults [1]. Most of the diseases associated with aging, such as those mentioned above, have a common characteristic in that they present a chronic and low-intensity neuroinflammation process [1]. In this process, there is an alteration in intracellular communication known as inflammaging, in which there is an increase in the secretion of proinflammatory cytokines and inflammatory markers [2]. Neurodegenerative diseases such as AD and PD have no cure, and in the case of FIS, therapeutic options are limited (thrombolytic drugs and mechanical thrombectomy), have a very short therapeutic window, are accessible to a small percentage of patients, and are not without dangerous side effects, so most patients with FIS only have the option of rehabilitation therapies [3]. On the other hand, the α-Klotho protein is well known for its anti-aging effects, and an extensive body of knowledge has been accumulated over several decades on its various beneficial effects, including reducing oxidative stress and neuroinflammation [4,5]. In the following sections of this paper, we briefly review relevant information about the α-Klotho protein, brain aging, AD, PD, FIS, neuroinflammation, and the modulation of neuroinflammation by α-Klotho in these diseases. Finally, we will discuss the therapeutic potential of this protein in AD, PD, and FIS.

## 2. Klotho Subtypes

In November 1997, Kuro-o and colleagues reported a new gene found in mice that was related to the suppression of several phenotypic features of aging, and they named this gene *klotho*. Defects in this gene produced several alterations similar to those occurring in humans, such as osteoporosis, atherosclerosis, infertility, and premature death [6]. Six years later, it was reported that overexpression of this gene produces a protein that, when released into the circulation, acts as a hormone and extends lifespan in mice [7]. Since the publication of these two very important articles, much progress has been made in understanding Klotho. Currently, three Klotho proteins are known, coded by different genes called *α*, *β*, and *γ* [6,8,9], all of which are transmembrane proteins that interact with different types of receptors to promote high-affinity binding of different members of the fibroblast growth factor (FGF) family [8,9]. α-Klotho interacts with the FGF23 receptor known as FGFR1c, which is highly expressed in the kidneys and brain and has been described as an anti-aging protein, while β-Klotho interacts with the same type of receptor expressed in adipocytes and with FGFR4 expressed in the liver, which are receptors for FGF21 and FGF19, respectively [10]. Meanwhile, γ-Klotho interacts with FGFR1c, 2c, and 4, which are expressed in the connective tissue, kidneys, and eyes, interacting with FGF19 [11]. The FGF family members interacting with the Klotho subtypes have different physiological effects. For example, FGF19 is a satiety hormone, as it activates metabolic responses in response to feeding; FGF21 is a hunger hormone that induces stress responses by activating the hypothalamic–pituitary–adrenal axis and the peripheral nervous system; and lastly, FGF23 is a hormone that increases the excretion of phosphate [10]. α-Klotho acts as a co-receptor to increase the affinity of FGF23, which modulates phosphorus and vitamin metabolism in the body, and the main consequence of its down-regulation is chronic renal failure [12,13]. In the following sections, we will only mention α-Klotho because it is the most studied in relation to aging and CNS diseases.

### 2.1. α-Klotho as a Hormone

**Figure 1:**
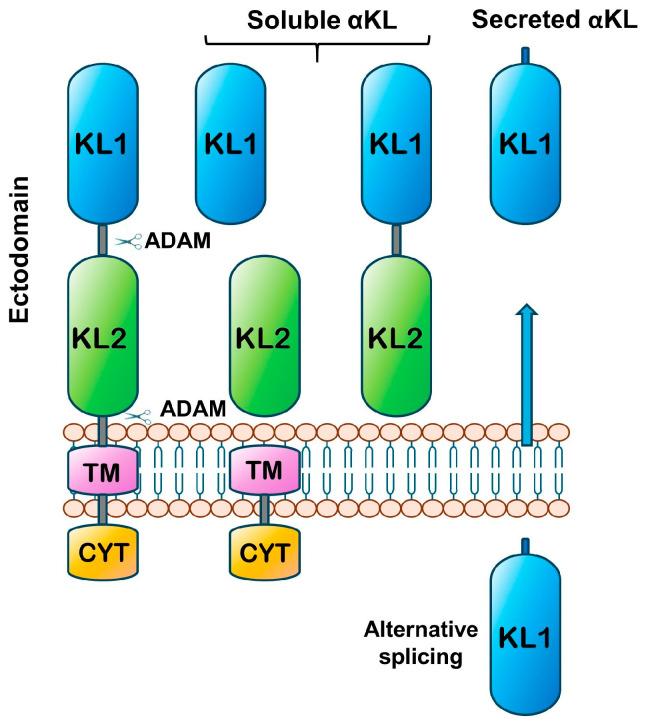
Klotho structure. The chart shows how the secreted and trimmed forms of α-Klotho are produced. The Klotho protein is membrane-bound and consists of three domains: a cytoplasmic domain (CYT), a transmembrane domain (TM) and an ectodomain. The latter is in turn formed by two internal repeats, KL1 and KL2. The ectodomain can be trimmed at two different levels by the enzyme.

The *α-klotho* gene is located on human chromosome XIII and has two major isoforms, a 135 KDa transmembrane protein that serves as an obligatory co-receptor for FGF23, and a 70 kDa secreted form [14]. α-Klotho is a single-step transmembrane protein, having a short intracellular domain and a long extracellular domain with two segments (KL1 and KL2) which are cleaved by enzymes (ADAM 10 and ADAM17) to produce three soluble fragments (Figure 1) that can circulate in the blood, cerebrospinal fluid, and urine [10,15,16]. Additionally, another isoform of α-Klotho can be produced by alternative mRNA splicing and includes the KL1 domain [17], which is sometimes called secreted Klotho or se-Klotho, whose functionality was previously doubted [18], but is now known to be fully functional [19,20,21]. This isoform is much more abundant (10 times more) in the brain than the cleaved isoform and has only been identified in the mouse brain [22]. There is evidence that both cleaved and secreted α-Klotho are found in human cerebrospinal fluid (CSF) and that the concentrations found in CSF and plasma have no correlation [23]. Together with the fact that α-Klotho does not permeate the blood–brain barrier (BBB), evidence suggests that α-Klotho present in plasma and CSF are independently regulated in the kidney and choroid plexus, respectively [24,25]. Hereafter, we will refer to the soluble and secreted isoforms simply as α-Klotho, unless it is an effect or function characterized in a particular protein isoform. The main source of α-Klotho in the blood comes from the kidneys [6,26,27]. α-Klotho performs many functions through a mechanism that is independent of FGF receptors and involves the modulation of sialic acid-containing cell membrane microdomains, such as monosialogangliosides GM1 and GM3 present in lipid rafts, which function as signaling hubs for trophic events [28,29,30]. Other studies have shown that α-Klotho regulates the activity of several ion channels, ion transporters, and growth factor receptors on the cell membrane surface. For example, it increases transient receptor potential cation channels, subfamily V, member 5 channels (TRPV5), and the ROMK1-mediated potassium current [31,32] in the cell membrane. In these channels, α-Klotho removes the sialic acid terminus of N-glycan, which prevents its endocytosis and thus increases its abundance in renal tubule channels and results in an improvement in Ca^2+^ and K^+^ regulation [33]. In the arcuate nucleus (ARC) of the hypothalamus, α-Klotho produces complex effects; in 44% of proopiomelanocortin (POMC) neurons, it produces excitatory effects (depolarizes and increases firing rate), and in 55%, it produces inhibitory effects (decreases membrane potential and firing rate) [34]. Additionally, there are many observational studies that establish a correlation between plasma α-Klotho concentration and different disorders and aging. For example, CSF α-Klotho concentrations are lower in older versus younger adults [35], and higher plasma α-Klotho concentrations were associated with a lower risk of meaningful decline and a smaller average decline according to the Mini-Mental State Examination [36]. Other studies have also shown that low plasma concentrations of α-Klotho in older adults are associated with cognitive function decline [37,38]. Low α-Klotho concentrations have also been associated with various diseases such as cardiovascular diseases [39], metabolic syndrome [40], and chronic kidney disease [41], and due to the inability of α-Klotho to cross the BBB, it has been suggested that the primary source is the kidneys since some studies show that this protein mainly comes from these organs [24]. Since α-Klotho concentration in blood plasma has a negative correlation with health status in observational studies of patients with various diseases including nerve diseases, it is thought to be a useful biomarker of health status. Some research groups actually propose to include it as a biomarker of aging and frailty [42]. Recently, a negative correlation has been found between plasma α-Klotho concentration and the SII or systemic immuno-inflammatory index [43], which provides a quantitative measure of the inflammatory immune response of the human body [44]. However, it is not known whether the association between blood α-Klotho levels and patient status is causal.

### 2.2. α-klotho Polymorphism

Within the human population, it has been found that the *α-klotho* gene has slight variations (mutations) in its DNA sequence (polymorphic gene), and several variants (alleles) have been found to vary by a single nucleotide (single-nucleotide polymorphism or SNP). It is known that these variations can influence the function of the protein. α-Klotho can be measured and studied by obtaining the protein present in blood serum and it has been used to study if there is any correlation between any polymorphism and longevity, cognition, or any disease. For example, the best studied polymorphism is the functional variant *KL-VS*, which contains six SNP sequence variants. A meta-analysis concluded that it is positively associated with exceptional human longevity [45]. However, in another study, two particular polymorphisms (*G-395A* and *F352V*) were examined and it was found that the *G-395A* polymorphism is associated with a high risk of urolithiasis and cardiovascular disease but not with cognitive decline, while *F352V* is associated with protection against breast cancer and longevity [46]. In another study, the *KL-VS* polymorphism (*rs95363147/g*) was associated with an increased incidence of dementia in adults [47]. Other research papers have found differences in their correlation with an elevated or reduced risk of disease, depending on whether the mutations are in both copies of the gene (homozygous genotype) or only in one (heterozygous genotype), e.g., homozygous *KL-VS* individuals are at a higher risk of stroke than heterozygous individuals [48]. Further studies are needed to more clearly establish the relationship between polymorphic variants of *klotho* and resistance or susceptibility to a specific disease.

### 2.3. α-Klotho in the Brain

α-Klotho is expressed in neurons of several brain regions such as the hippocampus, cortex, striatum, and Purkinje cells in the cerebellum, but the highest expression levels are in the choroid plexus cells [6,49,50]. Some of the functions of α-Klotho in the brain were elucidated in the hippomorphic *kl/kl* mice developed by Kuro-o, showing an aging phenotype [6], and in mice that overexpress α-klotho [7]. From the work with these animals, it can be concluded that the absence of the *klotho* gene causes disorders in axonal transport, a reduction in the number of synapses in the hippocampus, and neurodegeneration in the same tissue. Moreover, reduced Purkinje cells in the cerebellum were also reported. Meanwhile, overexpression of the gene produces an improvement in cognitive functions and, in particular, memory-related tasks, possibly by increasing the number of glutamatergic synapses in the hippocampus and cortex [51,52,53]. In addition, α-Klotho seems to exert a regulatory effect on oligodendrocytes and myelin, since it has been reported that in mice deficient in this protein, the number of oligodendrocytes is lower and myelin is also reduced, while overexpression of the gene produces an increase in myelin. In addition, there is a correlation between the decrease in α-Klotho and cerebral white matter lesions [54,55,56]. The brain functions regulated by α-Klotho are performed via the same mechanism mentioned above, i.e., by modulating sialic acid-containing microdomains of cell membranes such as monosialogangliosides GM1 and GM3 present in lipid rafts and regulating ion channels that can influence intracellular signaling and cell-to-cell communication [28,29,30].

## 3. Brain Aging and α-Klotho

### 3.1. Aging Brain

Aging is a complex and inevitable process that results from the interaction of environmental, epigenetic, and genetic factors that produce a gradual reduction in homeostasis with age [1]. This process is characterized by progressive tissue degeneration and a loss of function in various organs, which contributes to an increased risk of disease and death [57,58]. Aging affects individuals differently, but some changes occur more frequently. At present, twelve hallmarks are recognized in aging and these are interconnected with each other. These hallmarks are telomere attrition, genomic instability, epigenetic alterations, loss of proteostasis, cellular senescence, disabled macroautophagy, deregulated nutrient-sensing, altered intercellular communication, mitochondrial dysfunction, stem cell exhaustion, dysbiosis, and inflammation [59]. The aging human brain experiences both macroscopic and microscopic changes, including a decrease in white and gray matter density, thinning of the cortex, atrophic regions (e.g., hippocampus), enlargement of the ventricles, a decrease in blood flow, and a reduction in synapse and neurotransmitter release [60,61]. Diseases whose incidence increases with age are known as aging-related diseases and include neurodegenerative (e.g., AD and PD), metabolic (e.g., type 2 diabetes), cancer, and cerebrovascular diseases (e.g., FIS). Chronic low-intensity neuroinflammation is a feature of aging and is thought to be an important factor in amplifying damage in different diseases by accelerating neuronal dysfunction and death [62]. There are two aspects of brain aging that warrant a more detailed description. The first is cognitive dysfunction, as it represents a manifestation of an underlying disease and is common in many neurodegenerative diseases. The other is the physical barriers that separate blood from brain tissue and cerebrospinal fluid, due to their implications in the neuroinflammatory response, which will be described separately below.

#### 3.1.1. Aging-Associated Cognitive Impairment

Cognitive dysfunction describes the decline in a person’s mental abilities and is known to increase with advancing age [63]. Cognitive aging is one of the obvious signs of neurodegenerative diseases such as AD [64]. Cognitive functions include learning and memory, information manipulation, attention, perception, decision making, speech and language, and social cognition [65]. Cognitive aging is the result of anatomical and functional changes in different brain regions associated with a particular cognitive function, and the magnitude of the alteration is related to genetic and environmental factors (lifestyle, education, experience, emotional factors, health status, and socio-economic factors), which vary among individuals [65]. Chronic peripheral and central inflammation are characteristics of aging and are linked to cognitive aging. Different studies have shown that markers of inflammation present in blood and CSF are associated with increased cognitive decline and progression of neurodegenerative diseases, especially in older people [66,67,68,69]. In animal studies, high levels of proinflammatory cytokines in blood serum and brain parenchyma are associated with cognitive deficits, particularly learning and memory [70], and the use of neutralizing antibodies against IL-17A, a proinflammatory cytokine, improves memory and reduces neuroinflammation [71]. Similarly, the use of the inhibitor PLX3397 and minocycline, compounds that inactivate reactive microglia, improved learning and memory in an animal model of PD [72].

#### 3.1.2. Age-Related Dysfunctions of the Blood–Brain Barrier and Blood–Brain–Cerebrospinal Fluid Barrier

The BBB and blood–cerebrospinal fluid barrier (BCSFB) deserve special mention as they act as a physical barrier for the peripheral immune system, preventing immune cells and antibodies from entering the brain, which would eventually cause an excessive inflammatory response [73,74]. The BBB is a structure present in the network of cerebral capillaries, which is formed by endothelial cells associated by tight junctions, pericytes, astrocytes, and a basement membrane that functions as a highly selective filter that regulates the passage of substances between the blood and brain tissue [74]. The main functions of the BBB are as follows: (1) protection, which prevents harmful substances from entering brain tissue. (2) maintaining homeostasis, controlling the entry of essential nutrients such as glucose and amino acids and the exit of metabolic waste, as well as limiting the penetration of drugs used to treat CNS diseases such as neurodegenerative diseases and cancer [74]. The BBB also undergoes alterations during healthy aging, and these alterations are considerably aggravated when neuroinflammation occurs, as in neurodegenerative diseases such as AD, PD, and stroke, where there is degradation of endothelial cells, a decrease in proteins that form tight junctions (e.g., claudin, occludin), and increased permeability, which, combined with endothelial cell senescence, greatly contribute to damage to the BBB and neurovascular coupling dysfunction. All of these events result in an increase in proinflammatory cytokines (e.g., IL-1β, IL-2, IL-6, TNF), activation of microglia and astrocytes, leukocyte infiltration, and increased oxidative stress [75]. On the other hand, the blood–cerebrospinal fluid barrier (BCSFB) is a structure specialized in regulating the movement of substances between the blood and cerebrospinal fluid. It is in cerebral ventricles and is formed by the choroid plexuses, which form a barrier between the blood transported in the fenestrated capillaries and the cerebrospinal fluid [76]. In addition, the choroid plexuses produce cerebrospinal fluid and α-Klotho [73]. The choroid plexuses are made up of epithelial cells, fibroblasts, endothelial cells, and macrophages, the latter located in the stroma and epiplexus [77]. In aging and neurodegenerative diseases such as AD, this barrier is dysfunctional and its integrity is compromised, facilitating the migration of circulating immune cells to the CSF, which is a marker of neuroinflammation in age-related neurodegenerative diseases [76]. Few studies have investigated α-Klotho on cerebral microvasculature, and it has been shown that endothelial cells in the human cerebral microvasculature differ from those in other parts of the body; for example, they do not express the protein and do not show some responses to FGF-23 stimulation that are dependent on α-Klotho, such as cell proliferation and nitric oxide production [78]. On the contrary, there is evidence in mice that a decrease in α-Klotho causes BCSFB dysfunction, as it has been shown that a decrease in α-Klotho secretion in the choroid plexuses causes macrophage infiltration, an increase in proinflammatory mediators, and microglia activation [79]. In the following sections, we will briefly describe neuroinflammation in aging, the most common diseases associated with aging, and the relevance of neuroinflammation in these diseases, and lastly, we will analyze the modulatory effect of α-Klotho on this pathological process in each of these diseases.

### 3.2. Neuroinflammation

Neuroinflammation refers to the response of the innate immune system of the central nervous system (CNS) when tissue damage or infection is detected. Acutely, it is a response that promotes tissue recovery, but chronically, it can promote damage [80]. Microglia and astrocytes are the main cells of the immune response in the brain and maintain bidirectional communication. They perform different functions during neuroinflammation and can promote protective or damaging actions, depending on the context of the inflammation. The main immune cell in the CNS is the microglia and these cells continuously monitor the parenchymal environment; if tissue damage occurs, they are the first to respond [81]. The immune response begins when the damaged or dead cells present in the CNS of non-communicable diseases release molecules (damage-associated molecular patterns: DAMPs) that are recognized by a family of receptors present in the cells, known as pattern recognition receptors (PRRs), which include different subfamilies, one of which is the Toll-like (TLR) family [62,82]. DAMPs include several molecules such as cytoplasmic and nuclear proteins released in necrosis, cytokines of the interleukin 1 (IL-1) family, ATP, and proteins present in diseases such as AD (Amyloid β) and PD (α-synuclein) [83]. The different DAMPs are usually recognized by different subtypes of TLRs and some of these are widely expressed by microglia and astrocytes and to a lesser extent by neurons and oligodendrocytes [84]. The interaction of DAMPs with their respective receptors in the cells triggers the activation of an intracellular signaling cascade such as mitogen-activated protein kinase (MAPK), phosphoinositide 3-kinase/protein kinase B (PI3K/AKT), and the mammalian target of rapamycin (mTOR), which in turn leads to the activation of different transcription factors such as nuclear factor kappa-B (NF-κB), activator protein 1 (AP-1), nitric oxide synthase (iNOS), and cyclooxygenase-2 (COX-2). These transcription factors regulate the production of proinflammatory cytokines and other mediators of the immune response, forming a cellular protection mechanism, but if stimulus clearance fails, this response can become chronic and harmful [1,85]. In chronic inflammation, once PRRs are activated, microglia undergo morphofunctional changes (reactive microglia) that induce the release of proinflammatory cytokines such as interleukin 1β (IL-1β), interleukin 6 (IL-6), tumor necrosis factor α (TNF-α), and interferon γ (IFN-γ), stimulating astrocytes in the area. Similar to what occurs in the microglia, the activation of PRRs triggers a series of morphofunctional changes in astrocytes (reactive astrocytes) that, in turn, induce a second inflammatory response because these cells also release proinflammatory cytokines such as IL-1β, TNF-α, and IL-6 [86]. Importantly, the changes experienced by microglia and astrocytes following PRR activation are multiple and are intimately related to the particular microenvironment in which the cells are found; so, the changes experienced vary between different physiological stages and across diseases, and even during the progression of a particular disease [87,88].

### 3.3. Neuroinflammation in Aging (Inflammaging)

In aging, there is an alteration in intercellular communication known as inflammaging, which consists of a state of low-intensity inflammation with an increase in the secretion of proinflammatory cytokines and inflammatory markers, but this alteration generally does not initiate the process of neurodegeneration, though it does contribute to magnifying the disease and is believed to participate in neuronal dysfunction and death [81]. This state of chronic low-intensity inflammation is in addition to another important feature of aging, cellular senescence, which was initially referred to as permanent proliferative arrest and therefore included only proliferating cells, but more recently, it has been shown that except for proliferative arrest, most of the other features of senescence (DNA damage, mitochondrial dysfunction, increased level of senescence-associated beta-galactosidase (SA-β-gal), senescence-associated secretory phenotypes (SASPs), etc.) are present in post-mitotic cells such as neurons [89]. In some neurodegenerative diseases and FIS, senescent cells are found, and it has been proposed that this contributes to the pathology of these disorders [90]. This contribution can be illustrated by the mitochondrial dysfunction that occurs in senescent cells which induces the secretion of several DAMPs that, in turn, activate multiple inflammatory cascades in neighboring cells, causing a chronic inflammatory response [62]. Several in vivo and in vitro studies show that microglia show important changes that suggest a reduction in their ability to perform their homeostatic functions and also that they may be senescent, e.g., they show an exaggerated inflammatory response, secrete more ROS, and their phagocytic and chemotactic capacity is reduced [91,92,93,94]. Furthermore, in aging and also in neurodegenerative diseases and FIS, there are alterations in the BBB that provoke the infiltration of cells of the peripheral immune system, favoring tissue regeneration or increasing neuroinflammation. In light of this, it is important to point out the role of the adaptive immune system in aging and these diseases. Recently, it has become evident that the gut–brain microbiota axis plays a very important role in aging and neuroinflammation. Intestinal dysbiosis may cause damage to the intestinal epithelium and provoke an immune response in the gut with the consequent release of proinflammatory cytokines that may translocate into the circulation and promote a systemic inflammation that may contribute to inflammaging and damage to the BBB, which would favor the infiltration of immune cells and contribute to increasing neuroinflammation and neurodegeneration [95]. Research suggests that the microbiota plays a role in neuroinflammation in AD, PD, and FIS. For example, short-chain fatty acids (SCFAs) from the microbiota increased microglial reactivity and promoted amyloid-beta deposition in an amyloidosis animal model [96]. Intestinal dysbiosis was found in a PD model, and transplantation of microbiota from these animals to healthy animals resulted in motor dysfunctions [97]. Similarly, in a PD animal model, transplantation of the microbiota from PD patients increased disorders in the animals compared to those who received microbiota transplants from healthy people [98].

### 3.4. α-Klotho Effects in Brain Aging

Although α-Klotho is a protein with anti-aging effects, it is important to note that its expression decreases with age [99], and this decline is associated with poor cognitive performance. For example, in one study, it was found that in women and men with decreased Klotho, low blood serum α-Klotho levels were associated with poor performance on cognitive tests [38]. Decades ago, it was observed that overexpression of this protein increased longevity and improved health, while its inhibition caused an acceleration in the presentation of a phenotype associated with aging [6,100]. α-Klotho has a protective effect on cognitive decline [25,53], through modulation of different mechanisms, including increased glutamatergic synapses [53], decreased endoplasmic reticulum (ER) stress, apoptosis, and vascular senescence [101,102], increased antioxidant mechanisms [103], the promotion of autophagy [104], the elimination of misfolded proteins, increased oligodendrocyte maturation and myelination of the CNS [105], and reduced gliosis and neuroinflammation [106,107,108]. In the last process, α-Klotho decreases the activation of NF-κB, reducing the production of proinflammatory cytokines [106,108,109]. α-Klotho deficiency causes activation of the Wnt/β catenin signaling pathway, which contributes to the aging process and germ cell exhaustion [110,111]. In contrast, α-Klotho interacts with soluble agonists as an antagonist of this pathway and blocks their interaction with receptors in such a way that it acts as an antagonist of Wnt/β catenin signaling [112]. In senescence-accelerated transgenic mice (SAMP 8), α-Klotho upregulation improved memory deficits and decreased the PI3/Akt pathway [113]. In a recent article, treatment with α-Klotho in old non-human primates significantly improved memory [114]. Using cultured human astrocytes and endothelial cells, it was observed that the secretion of proinflammatory substances such as Activin A and IL-1α secreted by senescent cells decreased mRNA in these cells, and that senescent cells transplanted into young mice produced a significant decrease in α-Klotho in the brain, kidneys, and urine [115]. Using an in vitro model of neuroinflammation (stimulation with lipopolysaccharide or LPS), it was found that treatment with α-Klotho significantly decreased TNF-α and IL-6 production, reversed NF-κB activation in glial cells, and decreased neuronal death caused by the secretion of these substances [116]. Despite the relevance of BBB dysfunction in neuroinflammation, there are no studies investigating the direct effect of α-Klotho on the cerebral vasculature, However, there is evidence that exposure of peripheral blood mononuclear cells from AD patients exposed to α-Klotho reduces plasma levels of IL-1β, IL-6 and TNF-α [107], suggesting that increased peripheral α-Klotho may decrease the effects of peripheral inflammation and thereby reduce BBB damage and neuroinflammation. Furthermore, there is evidence in mice that a decrease in Klotho causes BCSFB dysfunction, as it has been shown that a decrease in α-Klotho secretion in the choroid plexuses causes macrophage infiltration, an increase in proinflammatory mediators, and microglia activation [79].

## 4. Main Neurological Disorders Associated with Aging

The most common aging-related diseases include FIS and incurable neurodegenerative diseases such as AD and PD. Neurodegenerative diseases are different neurological disorders where modifications occur in specific areas, primarily in the synapses and neuronal networks, and in some cases, abnormal proteins accumulate and lead to cell death. The most common neurodegenerative disorders include, in addition to AD and PD, Huntington’s disease, and amyotrophic lateral sclerosis, and they all share a characteristic with other diseases associated with aging, such as FIS, this being that they all present chronic inflammation [117].

### 4.1. Alzheimer’s Disease (AD)

AD is a disease resulting from neuronal loss and atrophy of large areas of the hippocampus and cerebral cortex manifested by memory loss and difficulties in communication, problem solving, and behavior [118]. AD is generally grouped into two types, familial (FAD) and sporadic (SAD), also called idiopathic. FAD accounts for about 5% of reported cases and is caused by dominant mutations in the gene coding for Presynilin 1 (*PSEN1*), Presynilin 2 (*PRESN 2*), and the beta-amyloid protein precursor (*APP*). SAD, on the other hand, represents the remaining 95% of reported cases and has no genetic cause. Another frequently used classification, based on the age at disease onset, is division into late onset (≥65 years of age), which includes mostly people with SAD, and early onset (<65 years of age), which is mostly FAD [118,119]. The underlying cause of AD is still unknown but it is associated with synaptic loss and the presence of senile plaques, which are the accumulation of insoluble forms of the amyloid-beta (Aβ) protein that form extracellular plaques, and also intracellular accumulation of neurofibrillary tangles (NTF), which are the abnormal accumulation of hyperphosphorylated tau protein filaments, resulting in a loss of cytoskeletal microtubules and tubulin-associated proteins [120]. Among the hypotheses proposed to explain the causes of the disease, the three most widely accepted are as follows: (1) the cholinergic; (2) the amyloid, and (3) the Tau hypotheses. The first hypothesis essentially states that cognitive deficit is due to severe neurodegeneration of the cholinergic innervation, while the amyloid hypothesis suggests that increased Aβ42/Aβ40 induces neurodegeneration and cell death, and finally, the Tau hypothesis proposes that fibrillary aggregates of misfolded NTF proteins accumulate intracellularly in neurons and spread to other cells in a prion-like manner and disseminate throughout the brain [118,121].

#### 4.1.1. Neuroinflammation in AD

Microglia and astrocytes play a very important role in the pathology of AD. For example, there is evidence that in the early stage of the disease, neuroinflammation is already present. It has been proposed that this process is present several decades before the onset of severe cognitive impairment and that it is strongly linked to the accumulation of Aβ and NTF [122]. Activated microglia are involved in the protection of neurons and the degradation of Aβ, but if they fail to clear plaques, they become chronically activated, resulting in the secretion of proinflammatory cytokines and may also contribute to promoting plaque formation, as shown in one publication, where pharmacological removal of microglia resulted in a significant reduction in plaque deposition and intraneuronal amyloid [123,124]. In postmortem studies of brains from AD patients, alterations in the morphology of microglia and increased immunoreactivity to ionized calcium-binding adaptor molecule 1 (Iba-1) were found, suggesting the presence of activated microglia [125]. Some PET studies in humans using markers for activated microglia such as 11PK11195 and 11 PBR28 [126,127,128] have found these cells in several regions (e.g., frontal, temporal, parietal, occipital, and cingulate cortices). Other studies have used these markers for activated microglia in combination with markers for Aβ (e.g., 18 F-flutemetamol) or Tau (e.g., 18 F-AV145) and found a convergence of signals of the association cortex [129].

#### 4.1.2. α-Klotho Effects in AD

In an animal model (APP/PS1) of AD, α-Klotho overexpression significantly decreased the percentage of Aβ plaques in the cortex and hippocampus and soluble Aβ1-40 and Aβ1-42, and also decreased NLRP3/caspase-1 inflammasome signaling [108]. In another study using the same animal model, *α-klotho* overexpression was found to decrease cognitive deficits, inhibit AKT and mTOR pathways, and decrease Aβ1-40 in the brain [130]. In another animal model (SAMP8), Ligustilide (a molecule extracted in Chinese medicine plants such as *Angelica sinensis*) was found to decrease severe memory deficits, AB1-40 accumulation, and neuronal loss, among other beneficial effects, and also increased *α-klotho* expression in choroid plexuses and serum [131]. Subsequently, this natural compound was also found to increase soluble APP processing and autophagy [132]. In a study conducted in people of both sexes and in a wide age range, it was found that α-Klotho levels in serum and CSF levels are positively correlated and that both levels are negatively correlated with clinical dementia rating [133]. In a study using MRI in elderly people with cognitive decline, reduced α-Klotho levels were found to correlate with brain lesions in deep white matter [54].

### 4.2. Parkinson’s Disease (PD)

PD is a multifactorial condition manifested by bradykinesia, resting tremors, and rigidity, and it shares these symptoms with other disorders that together are known as parkinsonism [134]. In PD, other disorders such as sleep disturbance, depression, and dementia are also observed [135]. The main microscopic features of PD are degeneration of the dopaminergic neurons of the substantia nigra pars compacta and intraneuronal protein aggregates, known as Lewy bodies [136]. Most PD patients correspond to the idiopathic type and very few cases are of genetic origin. In the latter type, several mutated genes are involved, such as α-synuclein (the main component of Lewy bodies), in which point mutations have been found in addition to gene duplication or triplication. On the other hand, in the idiopathic form, several risk factors have been identified, such as aging, exposure to pesticides, and traumatic brain injury [136]. In non-pathological conditions, α-synuclein has two conformations, a soluble unfolded monomer and membrane-bound multimeric α-helical form. α-synuclein is involved in various cellular functions in neurons, such as dopamine synthesis and vesicle trafficking. Under pathological conditions, the soluble unfolded monomeric form generates oligomers called protofibrils that transform into insoluble fibrils, which can spread from neuron to neuron via a transcellular mechanism [136]. In addition to the neurons of the substantia nigra, other structures such as the basal nucleus of Meynert, the raphe nuclei, the amygdala, and the neocortex also show Lewy body inclusions [137].

#### 4.2.1. Neuroinflammation in PD

The presence of neuroinflammation in the pathology of Parkinson’s disease is observed in various studies, such as postmortem or in vivo PET studies in patients. In postmortem studies, activated astrocytes and microglia have been found in the substantia nigra of brains of patients with the disease [138,139,140]. In a PET study, activated microglia were found to be present in several regions, including the substantia nigra, caudate nucleus, and frontal lobe [141]. Some studies using animal models and postmortem studies of patients have provided evidence for the possible involvement of the adaptive immune system; for example, T cells (CD4+ and CD8+) were found in the substantia nigra [142]. Some in vitro studies show that α-synuclein induces the activation of microglia and increases the production of TNF α and IL-1B [143]. α-synuclein induces the activation of the NF-κB signaling pathway in microglia and this signaling pathway leads to the activation of the NLRP3 inflammasome, which in turn promotes the release of IL-1β [144].

#### 4.2.2. α-Klotho Effects in PD

In *klotho*-deficient mice, a lower number of dopaminergic neurons are observed in the substantia nigra and ventral tegmental area of the brain, and in the same type of animals, but older, a striatal reduction in dopamine is observed [145]. In a transgenic mouse model overexpressing α-synuclein, involving both young and old mice, it was observed that I.P. application of an α-Klotho fragment produced a significant improvement in learning and memory tests, but this improvement was blocked by treatment with GluN2B subunit blockers of the NMDA-type glutamate receptor, although the mechanism by which this process occurs is not known [25]. In a different animal model of PD (Intrastriatal 6-OHDA-lesioned rats), pretreatment with α-Klotho produced a decrease in reactive oxygen species, GFAP, DNA fragmentation, and α-synuclein, among other indicators, thus collectively providing neuroprotection [106]. In a study of PD patients, high levels of α-Klotho were found to be associated with a slower onset of cognitive decline [146]. Furthermore, in a small study involving patients with PD, it was found that α-Klotho is decreased in blood serum but increased in cerebrospinal fluid, and in the latter compartment, α-synuclein is inversely related to α-Klotho, suggesting that α-Klotho in cerebrospinal fluid is associated with the neurodegenerative process and α-Klotho in serum is related to peripheral and systemic changes [147].

### 4.3. Focal Ischemic Stroke (FIS)

Stroke is the most common cerebrovascular disease. According to the World Stroke Organization, each year, approximately 12.2 million people suffer a stroke, half of whom may die, while survivors may experience severe impairment in functions such as mobility, language, and emotions. In addition, it also states that most cases occur between the ages of 50 and 70 [148]. There are two main types of stroke, hemorrhagic, which represents ~15%, and ischemic, which represents the remaining ~85%; within the latter type, the most common is focal ischemic infarction, which is caused by the occlusion of a cerebral artery from either the local formation of a thrombus or an embolus that originated distally [3]. Stroke can be caused by a number of factors but the most important are small vessel disease (including arteriosclerosis and cerebral amyloid angiopathy), cardioembolism, and large artery disease, caused by stenosis or the occlusion of a large artery in the brain [149].

Currently, there are two types of treatments for this condition, one is treatment with a fibrinolytic agent and the other is via mechanical removal. Both are effective, but their therapeutic window is very short, and they are not without side effects. Treatment with a fibrinolytic agent such as recombinant tissue plasminogen activator (rt-PA, alteplase) is effective but its therapeutic window is four and a half hours from the onset of stroke and there is a potential risk of hemorrhage. Mechanical removal, or endovascular thrombectomy, is also effective and its therapeutic window can be extended up to six hours from the onset of stroke. In both cases, it is necessary to evaluate patients and determine the suitability of the treatment options in order to minimize side effects. Generally, these treatments are only applied in places where trained personnel and imaging units are available [150,151].

The reduction or cessation of blood perfusion in a specific area of the brain decreases the oxygen and glucose supply necessary to meet metabolic demand and can cause different levels of damage depending on different factors, for example, the magnitude and time of the reduction in blood flow and the susceptibility of the damaged tissue. In general, two different areas are observed at the injury site, one necrotic and the other with viable tissue surrounding the necrotic zone (peri-infarct), in which different events take place such as excitotoxicity, ionic alterations, cytotoxic edema, peri-infarct depolarization, neuroinflammation, etc., which together constitute the ischemic cascade [3].

#### 4.3.1. Neuroinflammation in FIS

Excitotoxicity and other damage mechanisms harm and kill cells in the stroke zone, releasing DAMPs that eventually interact with their respective receptors on the different cell types present in the peri-infarct zone and within these cells. The first to respond is the microglia, which quickly move towards the damaged zone and release different proinflammatory cytokines (e.g., TNFα, IL-1α and C1q) to stimulate the generation of reactive astrocytes with a proinflammatory secretory profile; they also secrete other compounds (e.g., MMP-9) that contribute to the weakening of the BBB and facilitate the infiltration of leukocytes, exacerbating cell death. The weakening of the BBB continues, its permeability is further increased, and vasogenic edema occurs; the activated astrocytes release proinflammatory cytokines (IL-6, TNFα, IL-1α, IL-1β, IFN_ϒ_) and free radicals. Also, a subset of reactive astrocytes proliferate and together with other cell types (e.g., fibroblasts, microglia) begin to form the glial scar that delimits the area of injury and restricts the diffusion of neuroinflammation [3,152]. After stroke, neuroinflammation may persist for days or months, as long as tissue damage is not resolved, and if this is the case, the activated microglia that initially secreted anti-inflammatory cytokines undergo morphophysiological changes and secrete proinflammatory cytokines [3,152]. In recent years, evidence has been accumulating in humans and animals linking the development of cognitive disorders and neuroinflammation. Post-stroke secondary inflammation has been found at sites distant from the primary lesion but related to the altered function [153,154,155,156,157]. Cognitive disturbances or dementia are common among stroke survivors, and these disorders together occur in approximately 70% of patients [158,159]. The pathophysiology of these alterations is not well known, but it is known that several factors favor their presentation, including the severity of the stroke, the volume of the lesion, the site, and pre-existing diseases [160]. Post-stroke cognitive impairment (PSCI) commonly occurs during the first year and may be completely reversible in some cases (20%), but for the majority, complete recovery does not occur and even about 30% develop dementia within the first 5 years [161]. PSCI manifests heterogeneously and some of the common disturbances include selective attention, language learning, episodic memory, and memory impairment [162].

#### 4.3.2. α-Klotho Effects in FIS

As mentioned above, Klotho decreases with age and Klotho deficiency is known to be associated with vascular calcification, impaired angiogenesis and vasculogenesis, and loss of arterial wall elasticity, which contributes to increased blood pressure and cardiovascular diseases [6,163]. Endothelial dysfunction and increased arterial stiffness contribute to increased blood pressure, cardiovascular problems, and cerebral infarction [164]. A low concentration of α-Klotho is also associated with cerebral small vessel disease [165], vascular cognitive impairment [166], and poor outcomes after three months of acute stroke [167]. In an observational study, the *klotho KL:VS* variant was found to be associated with stroke in young adults in India [168]. In animal models, overexpression of *klotho* significantly improves neurobehavioral deficits [169,170] and inhibits the inflammatory response by activating the Nrf2 pathway, which decreases the activity of the NLRP3 inflammasome, causing decreased neuroinflammation [171], while it also improves endothelial function by increasing nitric oxide [172] and reduces infarct size in MCAO rats via a mechanism involving a reduction in aquaporin 4 (AQP4). In a mice model, α-Klotho decreased GFAP and Iba-1 immunoreactivity in the ischemic tissue, inhibiting RIG-I/NF-kB signaling activation and proinflammatory cytokine production [169]. Another study showed similar results, finding decreased reactive astrocytes and proinflammatory cytokines [170]. Some in vitro studies on endothelial cells showed that α-Klotho increased nitric oxide and the anti-oxidative effect by enhancing the expression of manganese superoxide dismutase (MnSOD) [173] and suppressing the proinflammatory cytokine TNF-α [174].

## 5. Discussion

Chronic neuroinflammation is present in AD, PD, and FIS and induces alterations ranging from the molecular level to the dysfunction of neuronal networks that manifest themselves in a decline in complex functions such as learning, memory, and neuroplasticity processes. AD and PD cannot be cured, and in the case of FIS, existing treatments have a very short therapeutic window and only a very small percentage of patients receive them; therefore, in most cases, they depend on rehabilitation therapy. In addition, post-stroke cognitive impairments are common and contribute significantly to increased mortality, permanent disability, and a decreased quality of life. Therefore, it is essential to continue the search for new compounds that can help reduce the harmful effects of these diseases.

α-Klotho has several beneficial effects on the brain, including decreasing oxidative stress, increasing synaptic plasticity, memory, and learning, and modulating the inflammatory response. The evidence that α-Klotho can decrease the inflammatory response comes from multiple sources and different approaches, such as overexpression of *α-klotho* in different animal models (AD, PD, etc.), direct administration in preclinic studies, and positive correlation in observational studies in humans. However, the mechanism of these effects is not fully understood. There is evidence that upregulation of *α-klotho* blocks important molecules in the development of the inflammatory response such as the inflammasome NLRP3 and the transcription factor NF-κB, which are involved in the production of proinflammatory cytokines. In different cell types, α-Klotho inhibits the translocation of NF-κB, and since this transcription factor is responsible for the expression of several proinflammatory proteins such as IL-1, IL-2, IL-6, IL-8, IL-12, and TNF-α, it inhibits their synthesis [174,175,176,177]. Moreover, it was also found to inhibit TLR4, and it is known that the activation of this receptor signals through NF-κB, and in this way, it could also inhibit inflammation [178]. α-Klotho inhibits NF-κB inflammasome activation and thus prevents the conversion of pro-IL-1β, pro-IL-18, and gasdermin D to their active forms, thereby inhibiting the proinflammatory effects of interleukins and pyroptosis, a type of gasdermin D-mediated cell death [108,179]. On the other hand, the synthesis and release of α-Klotho declines in aging, and because the aforementioned diseases occur mainly during aging, it is to be expected that α-Klotho levels in blood serum and CSF are decreased, as has been shown in several studies involving animal models and in observational studies with humans, where there is a negative association between plasma and CSF concentrations and cognitive impairment. Moreover, there is evidence that α-Klotho does not cross the BBB [24], so it is possible that this correlation reflects a decline in both peripheral and central α-Klotho responsiveness. However, experimental evidence suggests that there are several viable ways to increase α-Klotho synthesis and secretion, such as physical exercise, exogenous administration of α-Klotho, and some flavonoids and various approved drugs for different human diseases. We will discuss these options below, except for overexpression using molecular biological methods (e.g., gene editing) because it is not yet feasible to apply them to humans.

### 5.1. Age, Gender, and Serum α-Klotho

It is widely recognized that serum α-Klotho concentrations decrease with age in both sexes, and some studies show that adult women of all ages have higher concentrations than men [180,181]. However, there is a large variability in serum α-Klotho that is related to multiple factors that influence this variability such as physiological changes (e.g., hormonal fluctuations, Klotho metabolism), diseases, and lifestyle [181,182].

### 5.2. Physical Exercise and Exogenous Administration of α-Klotho

It is widely recognized that physical exercise, both aerobic and anaerobic, produces beneficial effects in animal models of neurodegenerative and traumatic diseases as well as in patients with these diseases. However, these benefits vary in relation to the many factors involved in each situation (age, gender, duration, frequency, type and intensity of training, health condition, exercise modality, etc.). Studies evaluating plasma α-Klotho show varied results, as do the protocols used, but for few exceptions [183], the results show a significant increase [184,185]. Aerobic exercise training (12 weeks) increases α-Klotho levels in sedentary middle-aged adults [185]. A single high-intensity physical exercise event produces a transient increase in α-Klotho levels in healthy volunteers [186]. A single maximal 20-min aerobic exercise session induces an increase in α-Klotho protein levels [187]. Acute physical exercise (45 min of treadmill running) significantly increases circulating α-Klotho in both young and old mice, although the response in aged animals is lower, and similar results have been observed in humans [188]. Twelve weeks of moderate aerobic exercise training increase plasma α-Klotho levels in postmenopausal women [189]. A meta-analysis concluded that, in general, physical training increases blood α-Klotho levels, but there is no consensus about which protocol produces the highest blood α-Klotho concentration [184]. In addition, within this type of study, some have found that there is a positive correlation between increased blood α-Klotho and cognitive improvement. Twelve weeks of training produced a significant increase in α-Klotho, brain blood flow, and hippocampus volume, and correlated with an improvement in episodic memory in older adults [190], but another study with a longer duration (26 weeks) in middle-aged adults at risk of AD found no significant increase in α-Klotho and no correlation with cognitive changes [183]. A simple explanation for this discrepancy could be due to the difference in age and duration of training between the studies, but both studies have a very small sample size, so more studies are needed to determine if physical exercise modifies cognition and elucidate the mechanisms involved. However, physical exercise increases plasma α-Klotho, and exogenous application of this protein has shown cognitive enhancement in preclinical models, suggesting that physical exercise may have modulatory effects on cognition. Administration of α-Klotho to young and old healthy rodents in a PD model significantly improved cognition [21,25]. In addition, subcutaneous injection of a single low dose of α-Klotho in old non-human primates significantly improved memory and this effect persisted for at least two weeks [114].

### 5.3. Repurposing Approved Drugs

Repurposing drugs refers to using drugs approved for one disease or target for a different therapeutic target. This strategy offers many advantages over the development of a new drug; for example, these drugs have already passed the development and clinical testing phases, so their approval for a new use would be faster and cheaper. Preclinical studies have shown that different statin (Atorvastatin, Pitavastatin and Simvastatin), drugs that regulate cholesterol (HDL and LDL) and triglycerides increase the expression of *klotho* in kidneys [191,192,193]. Combining the antileukemic drug Dasatinib and the flavonoid quercetin increases Klotho in the brain, kidneys, and in mice and humans urine [115]. It has been shown in clinical studies that some drugs used to treat diseases such as chronic renal failure and hypertension and whose mechanism of action is to block the renin angiotensin and aldosterone system (RAAS), such as losartan and valsartan, increase α-Klotho in blood serum [194,195,196]. Also, there are reports that PPAR_ϒ_ (peroxisome proliferator-activated receptor gamma) antagonists, pioglitazone and rosiglitazone, which are used to treat type 2 diabetes and metabolic syndrome, equally show an increase in circulating α-Klotho in animal models [197,198].

### 5.4. Other Compounds

Vitamin D supplementation and systemic use of the neurotransmitter GABA in a model of diabetes also produced an increase in α-Klotho [199,200]. In the kidney, vitamin D induces the synthesis of α-Klotho and fibroblast growth factor 23 (FGF23), and both proteins are involved in phosphate homeostasis [201]. Systemic treatment with GABA produces beneficial effects such as pancreatic β-cell regeneration in animal models of type 1 diabetes. One of the mechanisms found is through increased synthesis and secretion of α-Klotho, which decreases apoptosis, inflammation, and oxidative stress [202].

## 6. Research Limitations

The evidence on the neuroprotective and neuroplastic role of α-Klotho in neurodegenerative diseases and stroke is consistent; however, most of the evidence comes from preclinical studies and, to a lesser extent, from observational studies in humans. The anti-inflammatory effect of α-Klotho is one of the main beneficial effects but many basic aspects of α-Klotho modulation and immune response in the brain are unknown, and it is also unknown how increased peripheral α-Klotho is involved in the reduction in neuroinflammation. More observational studies in healthy humans and in humans with neurodegenerative diseases are needed to obtain a more solid picture of the relationship between peripheral α-Klotho, its genetic variants, and different neurodegenerative diseases and stroke.

## 7. Conclusions

**Figure 2:**
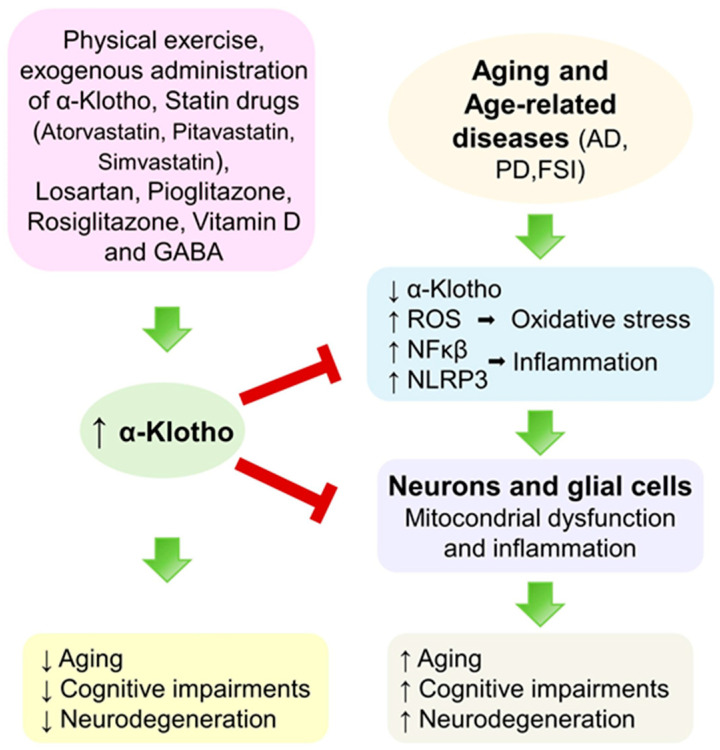
Different effects of α-Klotho increase in pathology of AD, PD and FIS.

Low levels of α-Klotho are associated with aging, cognitive impairment, and other disorders common to neurodegenerative diseases. α-Klotho augmentation has significant potential to improve brain function in older adults and in AD, PD, and FIS patients, probably through the activation of anti-inflammatory and antioxidant mechanisms, neurogenesis, synaptic plasticity, and cognitive functions related to the hippocampus (Figure 2). However, there are some basic unanswered questions, such as what signaling pathways modulate its synthesis, secretion, or inhibition in the brain, and how exogenous α-Klotho could participate in influencing brain physiopathology, among many other basic questions that need to be answered to allow us to better understand the physiology of this protein, which would help us to develop a therapeutic option.

## 8. Future Directions

Several biopharmaceutical companies are developing strategies to increase α-Klotho through gene therapy, such as the application of CRISP/Casp, but it is uncertain when this technology could be applied in humans. On the other hand, recombinant α-Klotho therapy is also very promising, but it is not yet available for use in humans and variants that can cross the BBB and exert their effects in the brain are yet to be found. Other strategies to increase endogenous α-Klotho production that have shown positive results should be further investigated, such as physical exercise, caloric restriction under medical supervision, the use of flavonoids, proopiomelanocortin, and the repurposing of some drugs. Finally, we can conclude that there are many possible therapeutic alternatives to treat neurodegenerative diseases and strokes, but much work remains to be done.

## Abbreviations

AD	Alzheimer’s disease
ADAM	a disintegrin and metalloproteinase
Apo	apolipoprotein
APP	amyloid precursor protein
BACE1	β-site amyloid precursor protein cleaving enzyme 1
BBB	blood–brain barrier
BCSFB	Blood–cerebrospinal fluid barrier
CNS	central nervous system
CP	choroid plexus
CSF	cerebrospinal fluid
DAMPs	damage-associated molecular patterns
Erk	extracellular signal-regulated kinase
FAD	familial Alzheimer’s disease
FGF	fibroblast growth factor
FGFR	FGF receptor
FoxO1	forkhead box protein O1
HEK	human embryonic kidney
IGF	insulin-like growth factor
IL	interleukin
KL	Klotho
KL-VS	Klotho with valine and serine substitutions
MHC II	major histocompatibility complex class II
MAPK	mitogen-activated protein kinase
MMSE	Mini-Mental Status Examination
NFκB	nuclear factor κ-light-chain-enhancer of activated B cells
NLRP3	NACHT-, LRR- and pyrin domain-containing protein 3
NMDA	N-methyl-d-aspartate
PERK	protein kinase R (PKR)-like endoplasmic reticulum kinase
PI3K	phosphoinositide 3-kinase
rDLPFC	right dorsolateral prefrontal cortex
PRRs	pattern recognition receptors
SAMP	senescence-accelerated prone mouse
SNP	single-nucleotide polymorphism
